# Editorial: New Insights on Neuron and Astrocyte Function From Cutting-Edge Optical Techniques

**DOI:** 10.3389/fncel.2019.00463

**Published:** 2019-10-15

**Authors:** Srdjan D. Antic, Bradley James Baker, Marco Canepari

**Affiliations:** ^1^Department of Neuroscience, Institute for Systems Genomics, Stem Cell Institute, UConn Health, Farmington, CT, United States; ^2^The Center for Functional Connectomics, Korea Institute of Science and Technology, Seoul, South Korea; ^3^Division of Bio-Medical Science and Technology, KIST School, Korea University of Science and Technology (UST), Seoul, South Korea; ^4^Laboratories of Excellence, Ion Channel Science and Therapeutics, Valbonne, France; ^5^Institut National de la Santé et Recherche Médicale, Paris, France

**Keywords:** optogenetic, voltage imaging, calcium imaging, GCaMP, voltage-sensitive dye

One of the most coveted goals of science is to elucidate the cellular bases of complex brain functions. How does a piece of biological tissue produce accurate cognitive insights, or succumbs to a devastating neurological disease? Neurons and astrocytes are the most numerous cells in the brain, comprising >95% of the brain parenchyma, and are responsible for all aspects of rapid information processing in the mammalian brain. Monitoring of physiological changes at the individual cell level, as well as in cell populations, is essential (Popovic M. et al., 2015; Nakajima et al., 2016). Early explorations of cellular physiology entailed metal (or glass and metal) electrodes. However, due to their highly invasive and tissue-harming nature, all electrodes, no matter how small, are limited experimental tools. While understanding brain circuits ultimately relies on the analyses of multiple and well-identified cells and subcellular compartments, the required information at high spatial and temporal resolution is not achievable using only electrode techniques for several reasons. First, the necessity for simultaneous measurements from ensembles of cells seems to be the most agreed upon aspect of the modern neuroscience (Barbera et al., 2016; Cai et al., 2016; Ji et al., 2016). Unfortunately, electrode arrays are limited in the number of cells they can monitor simultaneously. Second, the other reason for choosing optical imaging technologies over standard electrode recordings is that this is the only available approach to explore in detail thin processes of neurons and astrocytes, i.e., the essential cellular compartments underlying brain function (Larkum et al., 2018; Sheffield and Dombeck, 2019). In particular, in thin dendritic branches of neurons and astrocytes, optical measurements perform better than electrode recordings, in terms of calcium activity (Schiller et al., 1995; Kampa et al., 2006), membrane potential transients (Zhou et al., 2008), and also for recording ion currents (Jaafari et al., 2014; Jaafari and Canepari, 2016; Ait Ouares et al., 2019). In summary, the places where electrodes cannot go, optical measurements are taking over (Popovic M. A. et al., 2015; Bindocci et al., 2017; Gordleeva et al., 2019).

In the last decades, neuroscience research has been driven by the development of novel technologies interfacing scientists with molecules, cells, neuronal tissues, or the entire brain. Certainly, optical techniques have played a major role in this progress opening the gate to unprecedented information on living neurons and astrocytes. Innovative illumination and light recording strategies (Müllenbroich et al.; Ronzitti et al.; Battefeld et al.; Miyazaki et al.) advanced our knowledge on signaling in submicron structures (Francavilla et al.; Kuhn and Roome; Miyazaki et al.) while permitting the investigations in semi-intact tissues (Tominaga et al.; Kajiwara et al.; Quicke et al.). In parallel, the new emerging field of optogenetics allowed the selected expression of light-activated proteins that can be used for precisely targeted photostimulation (Baker and Flannery). Other optical strategies that recently progressed include combined Ca^2+^ and Na^+^ measurements (Miyazaki et al.), membrane potential recordings from individual cells (Francavilla et al.; Kuhn and Roome; Quicke et al.), or cell populations (Tominaga et al.; Kajiwara et al.; Li et al.), and chemical stimulation of targeted receptors using caged compounds (Palma-Cerda et al.).

## Studies Using Organic Indicators

In the early days of functional optical imaging, organic dyes were used for studying membrane potential changes in excitable cells and were dubbed voltage-sensitive dyes (Davila et al., 1973; Cohen et al., 1978). Later on, compounds were synthesized for measuring calcium or sodium transients in the cytosol (Brown et al., 1975; Grynkiewicz et al., 1985). Tominaga et al. have developed a voltage-sensitive dye imaging method for measuring compound synaptic potentials (population signals) from the entire hippocampus CA1 area for extended periods of time (experiments lasting up to 12 h). Their results shed light on a rarely discussed spatial aspect of long term potentiation (LTP) induction (e.g., magnitude of LTP increases with distance from the stimulation site) (Tominaga et al.). Using the same experimental approach, based on extracellular labeling of brain slices with voltage-sensitive dye and state-of-the-art wide-field single-photon voltage imaging at 2,000 frames per s, Kajiwara et al. showed that the efficacy of propagation of electrical activity between two critical brain regions, from perirhinal cortex to entorhinal cortex, is strongly dependent on membrane excitability and is malleable by synaptic plasticity (Kajiwara et al.). Miyazaki et al. have developed a robust method for simultaneous imaging of [Ca2^+^]_i_ and [Na^+^]_i_ changes in neurons at high speed and high spatial resolution. Via whole-cell electrode attached to the cell body, the authors co-inject two different pairs of spectrally orthogonal Ca^2+^ and Na^+^ indicators and excite the two dyes by illuminating at two wavelengths either using LEDs or laser spots. This method allowed them to monitor synaptically evoked Ca^2+^ and Na^+^ transients in individual dendritic spines (Miyazaki et al.). The review article by Dong et al. describes several techniques for studying gap junctions in the central nervous system. Fluorescent compounds and dyes are at the heart of several experimental approaches designed to probe the existence, strength, or nature of gap junction coupling: injection of small tracer molecules, scrape loading/dye transfer method, and recovery after photobleaching method (e.g., membrane-permeable fluorescein-AM). The review article by Kuhn and Roome, describes experimental design for performing dendritic voltage imaging in awake behaving animals. The authors also provide a very detailed account of the physical chemistry and quantum mechanics of charge-shift voltage-sensitive dyes with a special emphasis on two dyes from the Fromherz group (ANNINE-6 and ANNINE-6+). Physical chemistry and quantum mechanics of the organic dyes explains certain practical issues of working with fluorescent dyes, such as signal-to-noise ratio, phototoxicity, shape of the emission spectrum, dye flipping from one membrane leaf to another, etc. (Kuhn and Roome).

## Studies Using Genetically Encoded Indicators

Genetically encoded optical sensors of cell activity have become very popular in recent years, because they can be targeted to specific cell types. This is especially important in studies of the brain circuitry, because individual brain regions always include a multitude of different cell types, each type bestowed with a specific set of afferents and efferents, as well as rules for synaptic integration and patterned action potential firing. Francavilla et al. used viral transfection to label hippocampal GABAergic interneurons with the calcium indicator GCaMP6f. Two photon imaging of dendritic calcium transients (CaTs) was carried out in head-restrained mice running on a circular treadmill. Data showed that Ca^2+^ signals had larger amplitude and could invade the entire dendritic tree during locomotion, while during immobility, the signal amplitude was significantly lower in both soma and dendrites, and a significant fraction of dendrites showed spatially restricted CaTs, which were not seen in the soma. The authors proposed that strong dendrite-soma coupling, associated with animal locomotion, may facilitate the spike-timing-dependent or Hebbian forms of synaptic plasticity in interneurons. In contrast, during animal quiet state, this type of activity can be reduced or even replaced by local dendritic Ca^2+^ signaling, which may facilitate the anti-Hebbian plasticity mechanisms (Francavilla et al.). Another group working with GCaMP used the transgenic animal Thy1-GCaMP6f, expressing GCaMP6f in cortical pyramidal neurons (Li et al.). These authors showed that they can monitor sharp waves (SWs), carbachol-induced theta oscillations, and interictal-like spikes in brain slices. Furthermore, the authors claim that population GCaMP6f signals are fast enough for monitoring theta and beta oscillations (<25 Hz), which is an advancement for Ca^2+^ imaging. Quicke et al. also use a transgenic animal approach for delivery of a genetically encoded indicator to neurons. However, instead of GCaMP which is a calcium indicator, they use voltage indicator (VSFP Butterfly 1.2) to monitor membrane potential changes (Quicke et al.). Unlike the cytosolic molecule GCaMP, voltage indicators are membrane proteins (Kannan et al.). Their expression in brain tissue produces fluorescently labeled neuropil made of densely intermingled dendrites and axons, hence cellular resolution is lost. To achieve single cell resolution in voltage imaging, Quicke et al. combine recently developed sparse transgenic expression strategy (Song et al., 2017) and patch clamp recordings to demonstrate optical recordings of action potentials in individual neurons (Quicke et al.). Unlike GCaMP, genetically encoded voltage indicators have poor signal to noise ratio (Antic et al., 2016; Storace et al., 2016). But the signal-quality gap between GCaMP and GEVI is becoming narrower each year (Adam et al., 2019; Qian et al., 2019). A set of the state-of-the-art strategies for improving GEVI properties (sensitivity, brightness, SNR, fast kinetics, etc.) is described in an excellent article by Kannan et al., together with the overview of the pallet of currently available GEVIs (Kannan et al.). Finally, Storace et al. illustrate how membrane potential imaging using GEVIs (e.g., Arclight) can be utilized to decode the activity of the mammalian olfactory bulb, a goal that has been elusive for decades using older experimental approaches. Here, the authors used a clever strategy to simultaneously monitor input and output of a brain region in awake animal responding to controlled odor presentations. Afferent inputs to the olfactory glomeruli were assessed by calcium imaging from axon terminals of olfactory receptor cells (impinging onto the tufts of the mitral cells in the olfactory bulb glomerulus). Olfactory bulb outputs, on the other hand, were monitored by GEVI labeling of the mitral cells, whose axons project from the olfactory bulb into other brain regions. Simultaneous calcium and voltage imaging revealed that the output activity maps and the output signal size are much less concentration dependent than are the input maps and signals (Storace et al.). These results contribute immensely to our current understanding of the cellular mechanism for concentration invariance of odor recognition. The Storace et al. study is an excellent example of how applications of advanced imaging technologies (dual anatomical and genetic targeting leading to simultaneous calcium voltage imaging in an awake behaving animal) will impact systems neuroscience in the future.

## Advances of Optical Technologies

The ability of functional imaging to address fundamental questions depends on novel cutting-edge technologies and instrumentation. Battefeld et al. describe a versatile and open-source rapid LED switching system for one-photon imaging and photo-activation, that can be used either for quantitative ratiometric Ca^2+^ imaging or to combine Ca^2+^ imaging with optogenetic stimulation. Miyazaki et al. present an upgraded version of an imaging system, based on high speed multiplexing of light LEDs, to combine Na^+^ and Ca^2+^ imaging, a powerful approach to advance our knowledge on neuronal excitability and synaptic transmission. Ronzitti et al. review strategies for light shaping in three dimensions, including SLM-based multiplexing illumination, temporal focusing and other approaches. These rapidly developing illumination technologies open the gate to all-optical manipulation of neural circuits involving simultaneous stimulation of well-defined targets and simultaneous functional imaging of the resulting activity. Among light shaping approaches, light-sheet microscopy represents one of the most promising strategies. Using this technique, Müllenbroich et al. present an interesting improvement for imaging the transparent nervous system of zebra fish. In this application, Bessel beams are used to remove artifacts and dramatically improve sensitivity in Ca^2+^ imaging measurements (Müllenbroich et al.). Finally, a novel caged glutamate receptor antagonist is presented by Palma-Cerda et al. This compound combines the fast photolysis and hydrolytic stability of this family of photoactivatable molecules with fast-equilibrating competitive action on glutamate receptors, enabling deep investigation of postsynaptic functions at the molecular level (Palma-Cerda et al.).

## Author Contributions

All authors listed have made a substantial, direct and intellectual contribution to the work, and approved it for publication.

### Conflict of Interest

The authors declare that the research was conducted in the absence of any commercial or financial relationships that could be construed as a potential conflict of interest.
